# Baseline Serum C-Reactive Protein and Plasma Fibrinogen-Based Score in the Prediction of Survival in Glioblastoma

**DOI:** 10.3389/fonc.2021.653614

**Published:** 2021-03-04

**Authors:** Johannes Wach, Stefanos Apallas, Matthias Schneider, Agi Güresir, Patrick Schuss, Ulrich Herrlinger, Hartmut Vatter, Erdem Güresir

**Affiliations:** ^1^Department of Neurosurgery, University Hospital Bonn, Bonn, Germany; ^2^Division of Clinical Neuro-Oncology, Department of Neurology, University Hospital Bonn, Bonn, Germany

**Keywords:** fibrinogen, glioblastoma, score, survival, C-reactive protein

## Abstract

**Objective:** The present study investigates a score based on baseline C-reactive protein (CRP) and fibrinogen values (FC score) in 173 consecutive glioblastoma (GBM) patients.

**Methods:** The optimal cut-off value for fibrinogen and CRP was defined as 3.5 g/dl and 3.0 mg/L, respectively, according to previous reports. Patients with elevated CRP and fibrinogen were classified with a score of 2, those with an elevation of only one of these parameters were allocated a score of 1, and those without any abnormalities were assigned a score of 0.

**Results:** No significant differences in age, gender, tumor area, molecular pathology, physical status, or extent of resection were identified among the three groups defined by this score. Univariate survival analysis demonstrated that a high baseline FC score (≥1) is significantly associated with a shortened overall survival (OS) (HR: 1.52, 95% CI: 1.05–2.20, *p* = 0.027). A multivariate Cox regression analysis considering age (>65/≤65), extent of resection (GTR/STR), MGMT promoter status (hypermethylated/non-hypermethylated), and FC score (0/≥1) confirmed that an elevated FC score (≥1) is an independent predictor of shortened OS (HR: 1.71, 95% CI: 1.16–2.51, *p* = 0.006).

**Conclusions:** The baseline fibrinogen and CRP score thus serves as an independent predictor of OS in GBM. Further investigations of the role of inflammation in the prediction of a prognosis are needed.

## Introduction

Glioblastoma (GBM) is still the most devastating malignancy of the central nervous system. GBM represents 15.8% of all brain and CNS tumors (1). Established predictors for long-term survival in GBM include age at diagnosis, baseline Karnofsky Performance Status (KPS), and extent of resection (2–5). Furthermore, mutations in biological markers are important features to create a reliable prediction of the survival prognosis in GB. Hypermethylation of the O-6-methylguanine-DNA methyltransferase (MGMT) promoter, mutations in isocitrate dehydrogenase (IDH)-1 codon 132, and mutations of the promoter for the TERT gene are also independently associated with longer overall survival (OS) (6–9). Despite the prognostic benefits of evidence-based concomitant chemoradiotherapy regimens, including temozolomide (TMZ) and lomustine–TMZ, phase III trials reported that the median OS in patients with a hypermethylated MGMT promoter and treated with standard TMZ-based radiochemotherapy is 23.4–31.4 months (7, 10). Simple and inexpensive markers are required to create optimal adjuvant treatments and personalized follow-up schedules for patients with GBM.

Systemic inflammation is suggested to be associated with shortened survival in patients with cancer (11, 12). Fibrinogen is known to be a key biomarker in the regulation of inflammation, tumor cell proliferation, migration, and angiogenesis (13). Moreover, high levels of fibrinogen in the plasma are associated with shorter survival in a variety of cancers (14–17). Furthermore, C-reactive protein (CRP) was found to be an inflammation-related biomarker that can predict survival in GBM (18). However, C-reactive protein levels are not always elevated in patients with GBM, leaving the analysis of only one value in GBM insufficient for a reliable prediction of survival. For this reason, the fibrinogen and CRP score (FC score) was developed and found to be associated with survival in hepatocellular carcinoma and esophageal squamous cell carcinoma (19, 20). However, the predictive value of this score in glioblastoma is unknown.

The purpose of the present study was to assess the relationship between the preoperative fibrinogen and CRP score and survival among a homogeneous population of GBM patients who underwent surgical resection and started full radiochemotherapy.

## Methods

### Study Design and Patient Characteristics

**Figure 1:**
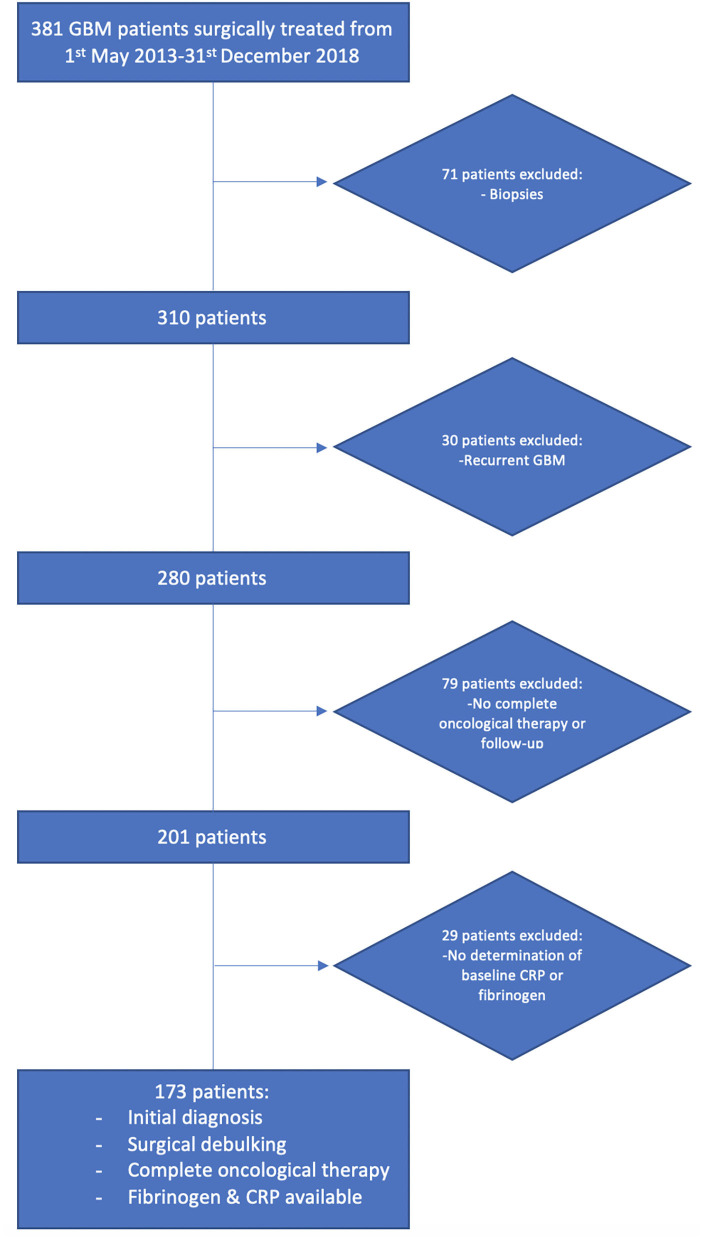
Flow chart illustrating the selection process of consecutive GBM patients between 2013 and 2018.

From January 2013 to December 2018, a total of 381 patients newly diagnosed with GBM were treated surgically at the Department of Neurosurgery, University of Bonn and analyzed retrospectively. The criteria for inclusion in this study were histopathologically confirmed GBM, an age >18 years, the availability of survival information and KPS, a single intracranial contrast-enhancing tumor lesion, treatment with neurosurgical resection via a craniotomy and surgical resection, and beginning post-operative radiotherapy and concomitant temozolomide chemotherapy or the first course of CCNU/temozolomide (10). Patients (*n* = 208) were excluded for the following reasons: only conventional stereotactic or VarioGuide (BrainLAB AG, Feldkirchen, Bavaria, Germany) biopsies without additional cytoreductive surgeries were performed, multiple intracranial lesions were present, the patients had partial or no clinical follow-up (≥1 month), the patients underwent surgery for recurrent GBM, and the patients had no availability of baseline serum CRP or plasma fibrinogen (Figure 1). A biopsy was performed if lesions were detected within the thalamus, internal capsule, splenium of the corpus callosum or brainstem; if an MRI revealed multiple or bilateral disease; or if the functional status was graded as KPS <60% (21).

### Preoperative Workflow

Routine blood investigations, blood coagulation tests, blood biochemical examinations, and head Gadolinium (Gd)-enhanced MRIs were routinely performed within 48 h before surgery.

### Surgical Procedure

White-light resection was performed under neuronavigation guidance (BrainLAB Curve, BrainLAB AG, Feldkirchen, Bavaria, Germany). When the surgeon believed that gross total resection (GTR) of the tumor was achieved, hemostasis was employed. Afterward, the resection cavity was examined using 5-aminolevulinic acid (5-ALA) (20 mg/kg, Gliolan; Medac GmbH, Wedel, Hamburg, Germany), and areas that were suspected to contain remaining tumor tissue were demarcated and resected. Post-operative MRIs were obtained within 72 h after surgery by a senior neuroradiologist to determine the extent of the resection (21). Gross total resection was defined as a resection without residual Gd-enhancement, whereas subtotal resection was considered any resection with residual Gd-enhancement and an extent of resection ≥90% (22).

### Histopathology

The histological evaluation was performed according to the World Health Organization 2016 diagnostic consensus criteria (23). For this purpose, paraffin sections were stained with hematoxylin and eosin (H&E). Sections were investigated immunohistochemically with Molecular Immunology Borstel-I (MIB-I) antibody, glial fibrillary acidic protein (GFAP), and IDH1 (21). MGMT status was and reported according to Hegi et al. (6). O-6-methylguanine-DNA methyltransferase promoter methylation was routinely assessed by pyrosequencing in 131 patients (75.7%) as described previously (24). For the categorization of tumors as either hypermethylated MGMT promoter or unmethylated MGMT promoter, we use a cut-off of <8% for the mean percentage of methylated alleles across CpGs for unmethylated MGMT promoters. In the present study 42 (24.3%) tumor samples were enrolled in the CeTeG/NOA-09 trial and analyzed with a quantitative methylation-specific PCR in central laboratories of MDxHealth (Herstal, Belgium) (10).

### Biochemical Measurements

Retrospective data acquisition was performed using the laboratory information system Lauris (version 17.06.21, Swisslab GmbH, Berlin, Germany). Venous blood samples were routinely collected within 24 h prior to the surgical resection of GBM. These laboratory examinations were performed at constant time points, which made it possible to analyze patient survival and progression rates. The standard examination before surgery included complete blood count, kidney, and liver tests. The coagulation profile (INR, aPTT) was also examined for every patient. The baseline plasma fibrinogen level was determined by the Clauss method, which involves adding a standard and high concentration of thrombin (Dade® thrombin reagent, Siemens Healthineers, Erlangen, Bavaria, Germany) to platelet poor plasma. This fibrinogen concentration is determined based on a reference curve. The serum C-reactive protein values were obtained by turbidimetric immunoassays with a CRPL3 reagent (Roche, Basel, Switzerland).

### Data Recording and Analysis

The following general preoperative characteristics of the patients were recorded: age, sex, Karnofsky Performance Status (KPS), American Society of Anesthesiologists (ASA), body mass index (BMI), and medical history of diabetes mellitus type II and secondary malignant neoplasms.

#### Tumor Characteristics

Tumor characteristics were analyzed based on a measurement of the midline shift, tumor area, maximum extent of the peritumoral oedema, and location classified according to the topography using the Sawaya grading system (25–29). Definitions and measurements of the features pertaining to tumor morphology were described in our previous study (21).

#### Fibrinogen and C-Reactive Protein Score

CRP was dichotomized into low (<3.0 mg/L) and high (≥3.0 mg/L) according to previous prospective data evaluating the baseline CRP in patients with cancer (30). A normal plasma concentration involves a fibrinogen level of 1.5–3.5 g/L. Hyperfibrinogenemia was defined as a plasma concentration >3.5 g/L according to the manufacturer's information and previous publications (31). Patients with both normal fibrinogen (<3.5 g/L) and CRP (<3.0 mg/L) were allocated a score of 0. Patients showing only one of these abnormalities were given a score of 1, while those with both abnormal CRP and fibrinogen were given a score of 2 (19).

### Follow-Up

Post-surgery treatment protocols were evaluated by the local tumor board review. Follow-up MRIs were routinely performed every 3 months (21). The Karnofsky Performance Status was assessed by neuro-oncologists at the follow-up examinations. Decisions and definitions of GBM progression were based on the Response Assessment in Neuro-Oncology (RANO) criteria, as actualized in 2017 (32). OS was defined as survival after the date of primary surgical resection in months.

### Statistics

We used the Fisher's exact test (two-sided) for nominal variables and Student's *t*-test for metric variables to compare the FC score groups. Only two-sided *p*-values were reported. Kaplan–Meier charts of OS and PFS were also calculated. Differences between the high (≥1) and low (0) FC score groups were analyzed using a log-rank test. A *p* < 0.05 was defined as statistically significant. Furthermore, a multivariate Cox regression analysis was performed to analyze the PFS and OS. Data were organized and analyzed using SPSS^©^ for MacOS 10.15 version 25.0 (IBM Corp, Armonk, NY, USA).

## Results

### Patient Characteristics

A total of 173 newly diagnosed GBM patients with available pretreatment fibrinogen and CRP records who underwent surgical resection and started post-surgical adjuvant therapy (radiotherapy ± alkylating chemotherapy) were analyzed. The proportions of patients with an FC score of 0, 1, and 2 were 60.7, 26.6, and 12.7% respectively. The median CRP and fibrinogen levels were 0.92 mg/L (range: 0.2–64.5) and 3.0 g/L (range: 0.4–5.5), respectively. The median age was 62 years (range: 18–85 years). Demographic parameters, physical status, body-mass-index (BMI), extent of resection [gross total resection (GTR), subtotal resection (STR)], MGMT promoter status, IDH-1 status, completion of post-operative treatment protocols, and type of treatment did not differ between the groups in terms of FC score (two-tailed Fisher's exact test). Comorbidities which are potential confounders with regard to the levels of inflammatory markers were also analyzed. Secondary malignant neoplasms, smoking, obesity, diabetes mellitus, connective tissue disorders, and blood-borne infectious diseases (HIV, Hepatitis B, and Hepatitis C) were homogeneously distributed among the FC-score groups. Further details of the baseline characteristics, established predictors, and treatment informations are summarized in Table 1.

**Table 1:** Clinicopathologic characters and fibrinogen–CRP score (Fisher's exact test).

**Characters**	**Fibrinogen–CRP score (Total** **=** **173)**			***p*-value**
	**0**	**1**	**2**	
	**(*n* = 105)**	**(*n* = 46)**	**(*n* = 22)**	
Sex (female/male)	40/65	17/29	6/16	0.68
Age (≤65/>65)	60/45	30/16	13/9	0.66
Secondary malignant neoplasm (present/absent)	11/94	1/45	2/20	0.23
Smoking (≥10 cigarettes/d) (yes/no)	9/73	7/32	2/15	0.54
Obesity (BMI ≥30.0) (present/absent)	15/86	8/36	4/17	0.79
Diabetes mellitus (present/absent)	7/98	5/41	4/18	0.18
Connective tissue diseases (present/absent)	5/100	4/42	2/20	0.48
Blood-borne infectious diseases (HIV, Hepatitis B/C) (present/absent)	0/105	1/45	0/22	0.39
Karnofsky performance status (<70/≥70)	4/101	0/46	1/21	0.40
Extent of resection (GTR/STR)	77/28	33/13	17/5	0.91
MGMT promoter status (hypermethylated/non-hypermethylated)	56/49	20/26	12/10	0.50
IDH1 status (mutated/wild type) available in 165 patients)	7/95	3/39	1/20	0.99
Completion of post-operative treatment protocols (full/preterm termination)	44/61	17/29	6/16	0.31
Radiotherapy alone (yes/no)	3/102	1/45	2/20	0.31

### Influence of FC Score on Overall Survival and Progression-Free Survival

**Figure 2:**
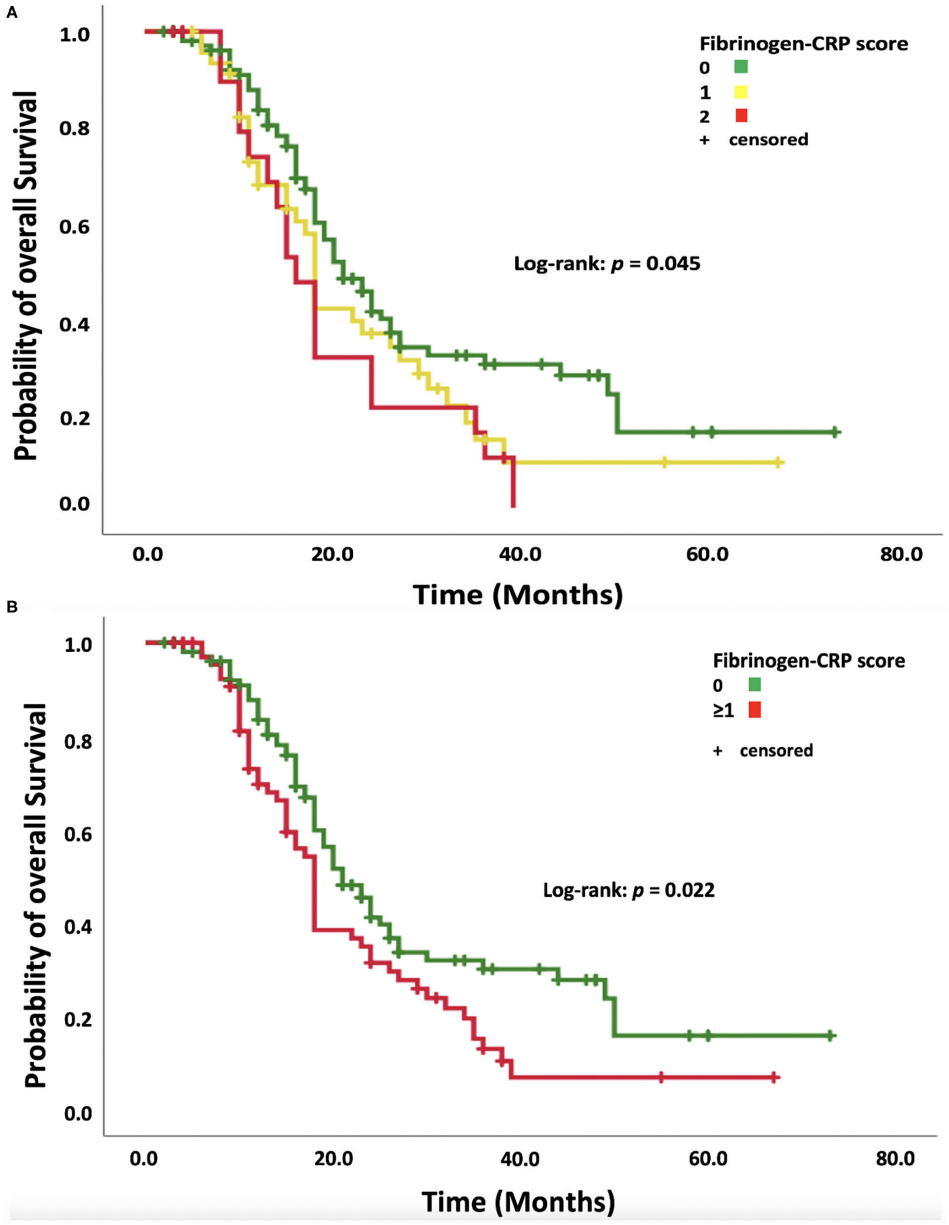
**(A)** Kaplan–Meier analysis of survival probability stratified according to fibrinogen–CRP scores of 0, 1, and 2. Censored patients (alive at last follow-up) are indicated on the curves. The time axis is right-censored at 80 months. *p* = 0.045 (log-rank test). **(B)** Kaplan–Meier analysis of the survival probability stratified by dichotomized fibrinogen–CRP scores of 0 and ≥1. Censored patients (alive at last follow-up) are indicated on the curves. The time axis is right-censored at 80 months. *p* = 0.022 (log-rank test).

The median OS for the entire cohort was 19.0 months [95% confidence interval (CI): 17.2–20.8]. Patients with an FC score of 0 had a median OS of 21.0 months (95% CI: 17.6–24.4), FC score 1 patients had a median OS of 18.0 months (95% CI: 17.0–19.0), and FC score 2 patients had a median OS of 16.0 months (95% CI: 13.2–18.8; log-rank test: *p* = 0.045, Figure 2A). Patients with an elevation of at least one systemic inflammatory parameter (FC score ≥1) had a median OS of 18.0 months (95% CI: 16.6–19.4, log-rank test: *p* = 0.022, Figure 2B). The median progression-free survival (PFS) was 9.0 months (95% CI: 8.0–10.0) in the entire cohort. The median PFS in the low FC score (0) group was 10.0 months (95% CI: 8.5–11.5), whereas patients with an FC score ≥1 had a median PFS of 9.0 months (95% CI: 8.5–9.5) (log-rank test: *p* = 0.27).

### Survival Comparison Between the Combined Systemic Inflammatory Parameters and CRP Only

**Figure 3:**
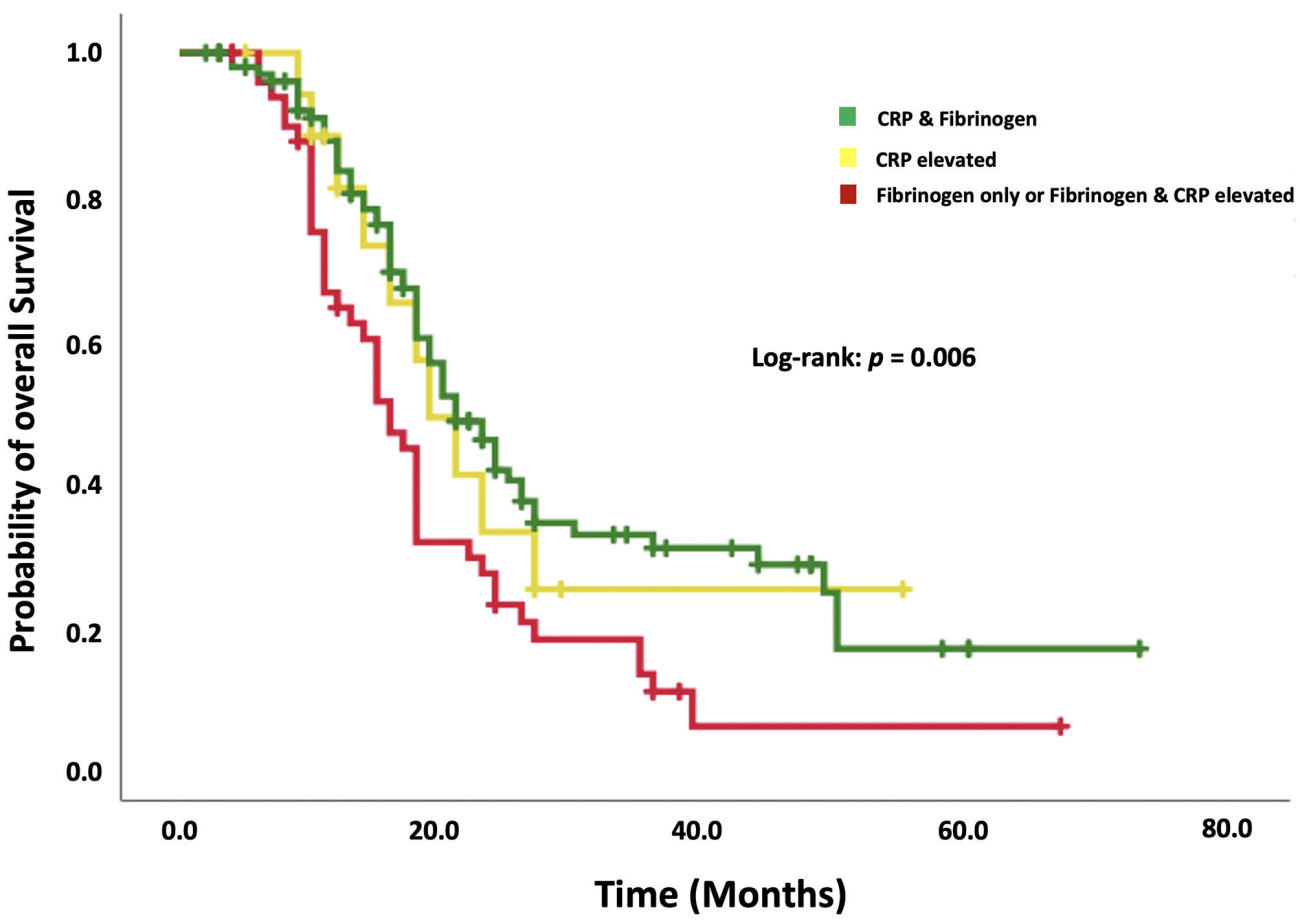
Kaplan–Meier analysis of the survival probability stratified by “normal CRP and fibrinogen,” “increased CRP only,” and “increased fibrinogen with or without increased CRP.” Censored patients (alive at last follow-up) are indicated on the curves. The time axis is right-censored at 80 months. *p* = 0.006 (log-rank test).

The median OS in patients without any increased acute-phase protein (*n* = 105) was 21.0 months (95% CI: 17.2–20.8). Eighteen patients featured only an elevation of CRP. The median OS in this subgroup was 19.0 months (95% CI: 13.9–24.0). Patients with increased fibrinogen combined with or without increased CRP (*n* = 50) had a median OS of 16.0 months (95% CI: 17.5–24.4). Figure 3 shows a Kaplan–Meier curve illustrating the superiority of a combined examination of fibrinogen and CRP compared to CRP only.

### Uni- and Multivariate Cox Regression Analysis of Overall Survival and Progression-Free Survival

A univariate Cox regression analysis of OS was performed for known predictors of OS in GBM and the FC score in the entire cohort. An age of >65 at diagnosis, a baseline FC score ≥ 1, subtotal resection, and a non-hypermethylated MGMT promoter status were significantly associated with shortened OS. We conducted a multivariate Cox regression analysis of OS considering age (>65/≤65), FC score (≥1/0), KPS (<70/≥70), extent of resection (STR/GTR), and MGMT promoter status (non-hypermethylated/hypermethylated). The multivariate Cox regression analysis identified age >65 (HR: 2.17, 95% CI: 1.46–3.23, *p* < 0.001), non-hypermethylated MGMT promoter status (HR: 3.09, 95% CI: 2.05–4.66, *p* < 0.001), and FC score ≥1 (HR: 1.71, 95% CI: 1.16–2.51, *p* = 0.006) as significant and independent predictors of shortened OS (Table 2). The univariate Cox regression analysis of PFS showed a HR of 1.20 in patients with a high FC score (≥1), which indicates a shortened time to tumor progression (95% CI: 0.84–1.73, *p* = 0.317). The multivariate Cox regression analysis of PFS with consideration of the FC score (≥1/0), age (>65/≤65), extent of resection (STR/GTR), and MGMT promoter status (non-hypermethylated/hypermethylated) identified only an unmethylated MGMT promoter (HR: 2.5, 95% CI: 1.7–3.8, *p* < 0.001) as an independent predictor of shortened PFS.

**Table 2:** Cox regression analysis of overall survival.

**Variable**	**Univariate**			**Multivariate**		
	**HR**	**95% CI**	***p*-value**	**HR**	**95% CI**	***p*-value**
Age (>65 vs. ≤65)	1.65	1.15–2.35	0.006	2.17	1.46–3.23	<0.001
Fibrinogen–CRP score (1 and 2 vs. 0)	1.52	1.05–2.20	0.027	1.71	1.16–2.51	0.006
Karnofsky performance status (<70 vs. ≥70)	1.26	0.40–4.0	0.69	1.47	0.45–4.80	0.53
Extent of resection (STR vs. GTR)	1.58	1.05–2.40	0.03	1.36	0.88–2.10	0.17
MGMT promoter status (non-hypermethylated vs. hypermethylated)	2.68	1.82–3.94	<0.001	3.09	2.05–4.66	<0.001
Gender (male vs. female)	1.17	0.80–1.71	0.42			NI
IDH-1 (wild-type vs. mutation)	1.22	0.57–2.63	0.60			NI

### Primary Stratification Tree and Overall Survival

**Figure 4:**
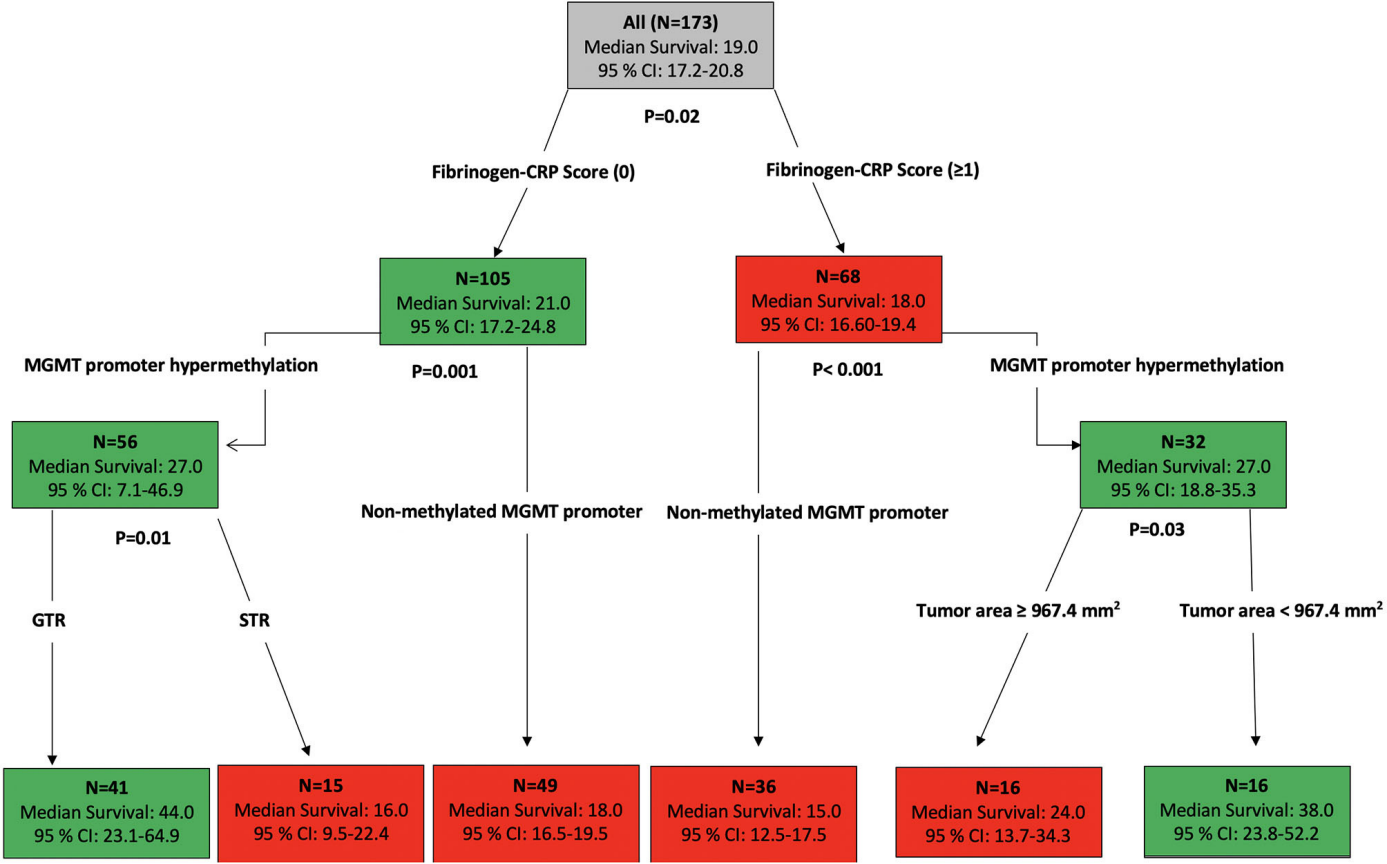
Primary stratification tree that presents the cohorts prognoses. Each colored box represents the specified subgroup with the number of patients, median OS in months, and 95% confidence interval. The green boxes indicate the subgroups of patients with good survival prognosis, whereas the red boxes represent the patients with poor prognosis. The entire patient cohort was first divided into fibrinogen–CRP score (0) vs. fibrinogen–CRP score (≥1), which showed an increase of 3 months in overall survival among patients with an FC score of 0 compared to patients with an FC score ≥1. The hypermethylated MGMT promoter status is an independent predictor of prolonged survival in both groups. The additional stratification of patients with an FC score of 0 whose MGMT promoters were hypermethylated revealed that the GTR in these patients increased their OS to 44 months (95% CI: 23.1–64.9), whereas further stratification of the patients with an FC score ≥1 whose MGMT promoters were not hypermethylated revealed a median OS of 15 months (95% CI: 12.5–17.5).

Using the data analysis presented above, an overall survival stratification tree was constructed and is shown in Figure 4. Each node in this tree summarizes the number of patients in the group, the median overall survival in months, and the two-sided 95% CI. At each node split, a univariate two-arm log-rank test was performed and displayed. Focusing on the high FC score (≥1) group (*n* = 68), 32 patients had MGMT promoters that were hypermethylated and a median OS of 27.0 months (95% CI: 18.8–35.3) compared to the 32 patients who had MGMT promoters that were unmethylated and a median OS of 15.0 months (95% CI: 12.5–17.5). After stratifying patients whose MGMT promoters were hypermethylated, those with a tumor area <967.4 mm^2^ had an increased median OS of 38.0 months (95% CI: 23.8–52.2). By stratifying patients whose baseline FC scores were low (0), those with a hypermethylated MGMT promoter were shown to have an increased median OS of 27.0 months (95% CI: 7.1–46.9). The addition of the criterion “GTR” to this group of patients showed an increase of 17.0 months in the median OS.

### Clinical Outcome

**Figure 5:**
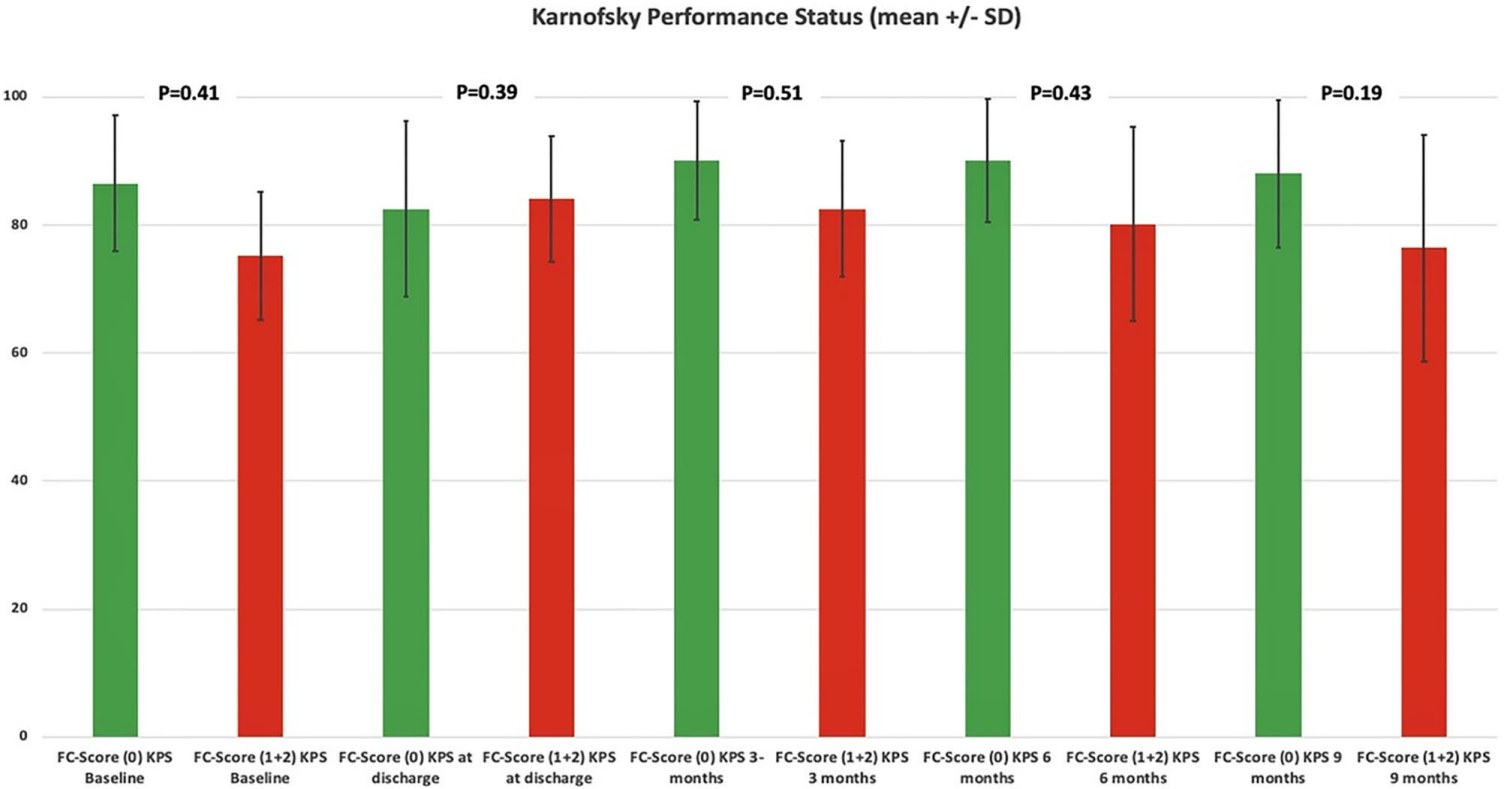
Functionality according to the Karnofsky Performance Status among dichotomized FC scores. Means (column) and 95% confidence intervals (lines) for the Karnofsky Performance Status, illustrating the degree of functionality in the period from admission to follow-up examination at 9 months stratified by the parameters FC score 0 (green column) and FC score ≥1 (red column). *p*-values of the Student's *t*-test are shown.

KPSs were homogeneously distributed among both the FC score groups (0 vs. ≥1). Patients with a high FC score ≥1 had a slightly poorer KPS at their 3-, 6-, and 12-months follow-ups. Statistical significance was not observed in the Student's *t*-test. Figure 5 displays the course of the KPS from baseline to the 9-month follow-up examination. The slightly poorer course of functional outcome in patients with a high FC score ≥ 1 did not significantly influence the adherence to post-operative treatment protocols. Forty-four (44/105; 41.9%) patients with a FC-score of 0, 17 (17/46; 37.0%) patients with a FC-score of 1 and 6 (6/22; 27.3%) completed all cycles of the scheduled treatment protocols, respectively [Fisher's exact test (two-sided), *p* = 0.31].

## Discussion

In this study, we evaluated the FC score as a prognostic dual biomarker score for GBM patients. We observed significantly decreased overall survival times in patients with an increased FC score (≥1) at the time of GBM diagnosis. An elevated FC score can be measured via determination of the plasma fibrinogen level and the serum CRP level prior to surgery for GBM and was found to be an independent risk factor for shortened survival. Our findings suggest the potential importance of assessing the prognosis of GBM by combining clinicopathological characteristics with initial inflammatory status.

In our cohort, 26.6% of patients presented with elevated fibrinogen plasma concentrations, and 12.7% of patients presented with elevated CRP serum concentrations, indicating that increased circulating concentrations of classical inflammatory response elements are common among GBM patients. There is emerging evidence that inflammatory response is associated with clinical outcomes in patients with various cancer entities, such as non-small cell lung cancer, ovarian cancer, and glioma (33–43). The FC score was first described in a retrospective study investigating 260 patients with esophageal squamous cell carcinoma. Univariate and multivariate analysis showed that high preoperative FC scores (≥1) were significantly associated with impaired disease-free survival and OS in patients with esophageal squamous cell carcinoma (20). Similarly, a retrospective study investigating recurrence-free survival and OS in 768 patients with hepatocellular carcinoma found a significant association with the FC score in the multivariate analysis (19). In our study, the FC score was also significantly associated with OS outcomes in GBM patients. Multivariate analysis with consideration of known predictors such as age, Karnofsky Performance Status, extent of resection, and MGMT promoter status confirmed that the FC score is an independent predictor of OS (3, 5, 6, 44). There are some potential mechanisms for the malignant impact of fibrinogen in gliomas, such as the relationship between inflammation and fibrinogen (45). Fibrinogen can activate leukocytes via integrin α Mβ2 (46). The neutrophil subpopulation of leukocytes secretes arginase-1 and vascular endothelial growth factors for immunosuppression and angiogenesis in gliomas (47). Furthermore, the function of natural killer cells was suggested to be negatively influenced by fibrinogen, which makes such cells unable to kill cancer cells (45). A retrospective study evaluating the plasma fibrinogen levels in 315 patients with GBM found that the plasma fibrinogen levels were significantly higher in IDH-1 wild-type GBMs with ATRX expression. Additionally, these patients had a significantly shorter OS compared to other patients (48). However, it has to be reminded that plasma fibrinogen levels can be influenced by the intake of drugs or the nutritional status (49, 50). Due to the common link of both C-reactive protein and fibrinogen to the interleukin-6 gene promoter, those confounding effects such as nutritional status or obesity also influence the C-reactive protein level (51). Therefore, a dual-biomarker approach assessing both fibrinogen and C-reactive protein may be more precise for a sufficient risk stratification of survival in GBM.

CRP is an established non-specific acute-phase protein that is synthetized in the liver (52). Several epidemiological studies have suggested that increased serum levels of CRP are associated with poor outcomes (53). Additionally, a meta-analysis found that the CRP value is associated with glioma risk, as well as with a poor prognosis (54). A retrospective analysis of CRP levels in 565 GBM patients also found an independent and significant role of CRP ≥2.0 mg/dl in the prediction of OS and 1-year survival (18). Some possible mechanisms elucidating the increased risk and poor prognosis of high CRP levels in gliomas have been reported (55). CRP acts on the microglia through IL-1β, which protects endothelial cells from starvation-induced death and thereby contributes to tumor angiogenesis and progression. Furthermore, interleukin-6 is secreted by GBM, which acts on CRP secreting hepatocytes and is caused by the Janus-kinase-signal transducer and activator of transcription (JAK-STAT) pathway. This secretion reaches the tumor site via the blood circulation and can accumulate in the tumor tissue. Cyclooxygenase-2 (COX-2) is also known to be an enzyme that is largely responsible for causing inflammation. GBM cells with COX-2 overexpression showed greater migration potential, and tumors that arose from these cells displayed increased microvessel density in line with increased malignant potential *in vivo* (56). Furthermore, it was found that the regular use of NSAID or COX-2 inhibitors resulted in a 33% reduction in the risk of glioma (57). The role of COX-2 was also investigated with regard to surgical resection and radiotherapy. It was found that the concentration of plasma prostaglandin E2 can be significantly reduced after the surgical removal of malignant gliomas (58). The COX-2 inhibitor celecoxib might play a critical role in the regulation of the growth of CD 133(+) glioblastoma stem-like cells. CD 133(+) GBM stem-like cells overexpressing COX-2 and celecoxib combined with radiation had a radiosensitizing effect in a mice model (59). However, the current limited evidence of clinical trials using celecoxib therapy in oncological studies is due to the known increased risk of myocardial infarction with celecoxib therapies (60).

The combined role of dual (fibrinogen + CRP) inflammatory biomarker screening in GBM has not yet been described. The baseline FC score ≥ 1 was significantly associated with a shortened OS, especially in patients with an unmethylated MGMT promoter. The stratification of patients for overall survival by tree-structured investigation revealed that the FC score ≥1 does not predict survival in patients with a hypermethylated MGMT promoter. Hübner et al. (61) found that miRNA-93 is a potential “anti-inflammatory tumor suppressor” in GBM and significantly decreases the expression of interleukin-6, interleukin-7, interleukin-1β, leukemia inhibitory factor, granulocyte-colony stimulating factor, COX-2, and CXCL5 *in vitro*. Additionally, the authors performed a TCGA analysis which confirmed that miRNA93 expression is negatively correlated with those mentioned target genes in human samples. Their survival analysis revealed that survival for tumors expressing a high level of miRNA93 is longer compared to a lower expression of miRNA93 in GBM. Interestingly, this finding was only significant in GBMs with a methylated MGMT promoter which suggests an important anti-inflammatory role of miRNA93 expression as a tumor suppressor in GBM patients with hypermethylated MGMT promoters.

MGMT promoter hypermethylation was found to be the only independent predictor for prolonged PFS in our multivariate cox regression analysis. One reason for this result might be the smaller number of patients with increased serum CRP or plasma fibrinogen levels compared to GBM patients with normal inflammatory parameters. Secondly, there was a slightly poorer clinical course among the patients with a high baseline FC score (≥1), which was reflected by a decrease of the KPS in our data. Therefore, the shorter OS, despite having no statistically significant association with PFS in the high FC score (≥1) group, might be explained by the poorer functional status that was reflected over the course of the KPS at the follow-up examinations in this GBM group. Furthermore, patients with a decreased and non-ambulatory functional status often do not pass the regular magnetic resonance imaging follow-up examinations as frequently as ambulatory patients with a good KPS. This bias might also influence the analysis of tumor progression and is a frequent limitation of retrospective oncological studies.

## Limitations

The present study has several limitations. Patient data were acquired retrospectively. The number of patients included in the high FC score (≥1) group was low because of the short time period that was chosen for analysis. However, this time period was chosen to rule out a lack of data on established prognostic molecular markers, such as IDH mutations and the MGMT promoter status in the cohort. However, we used highly selective inclusion criteria, such as an analysis of patients who underwent surgical resection and started post-operative chemo- and radiotherapy for primary GBM, to achieve a reliable analysis. Furthermore, the present investigation represents a single-center experience only. Despite the limitations, the results showed that the baseline FC score could be a fast and potential low-cost biomarker to predict overall survival and facilitate sufficient risk stratification and individualized interdisciplinary treatments for GBM patients. However, future investigations are needed to validate these findings in a large cohort study.

## Conclusions

FC score is a potential biomarker that can independently predict overall survival in GBM. Our investigation emphasizes the need for further studies evaluating the role of inflammation in the prognosis of GBM.

## Data Availability Statement

The raw data supporting the conclusions of this article will be made available by the authors, without undue reservation.

## Ethics Statement

The studies involving human participants were reviewed and approved by Ethics committee of the Rheinische Friedrich-Wilhelms-University, Bonn, Germany. Written informed consent for participation was not required for this study in accordance with the national legislation and the institutional requirements.

## Author Contributions

Data acquisition was performed by SA, MS, and JW. Study design, methodology, statistical analysis writing, and creation of figures were performed by JW and EG. Supervision of the study was done by EG. Proofreading was done by AG, PS, UH, EG, and HV. All authors contributed to the article and approved the submitted version.

## Conflict of Interest

The authors declare that the research was conducted in the absence of any commercial or financial relationships that could be construed as a potential conflict of interest.
