# Supplementary figures and images for: Baseline Serum C-Reactive Protein and Plasma Fibrinogen-Based Score in the Prediction of Survival in Glioblastoma
Source: Front Oncol. 2021 Mar 4;11:653614. doi: 10.3389/fonc.2021.653614 (PMC7970301; doi:10.3389/fonc.2021.653614)

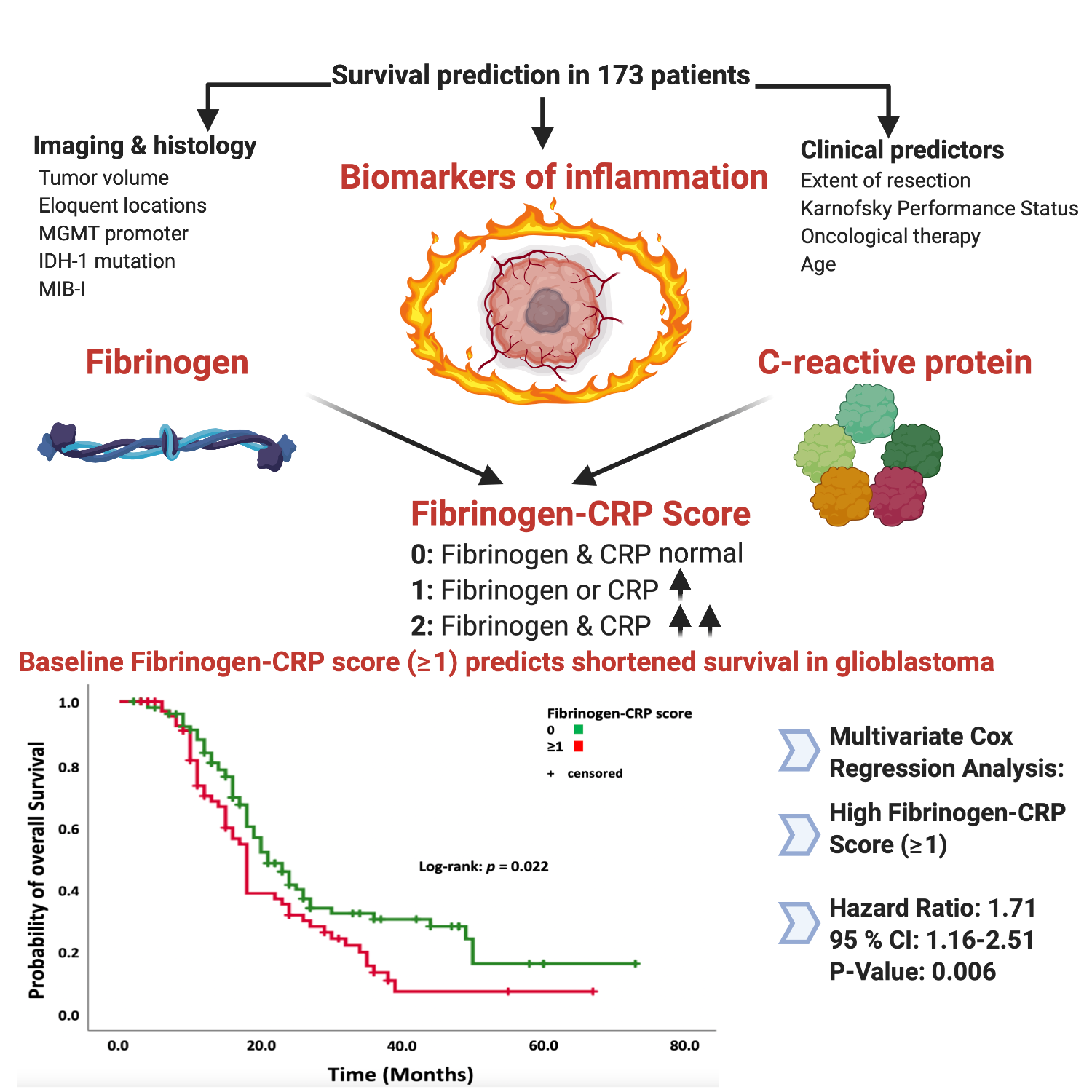

Supplement: Supplementary file 1 [file Image_1.TIFF]
